# Tinnitus: A Large VBM-EEG Correlational Study

**DOI:** 10.1371/journal.pone.0115122

**Published:** 2015-03-17

**Authors:** Sven Vanneste, Paul Van De Heyning, Dirk De Ridder

**Affiliations:** 1 Department of Translational Neuroscience, Faculty of Medicine, University of Antwerp, Antwerp, Belgium; 2 School for Behavioral & Brain Sciences, University of Texas at Dallas, Dallas, Texas, United States of America; 3 ENT Department, University Hospital Antwerp, Antwerp, Belgium; 4 Department of Surgical Sciences, Dunedin School of Medicine, University of Otago, Dunedin, New Zealand; University of Regensburg, GERMANY

## Abstract

A surprising fact in voxel-based morphometry (VBM) studies performed in tinnitus is that not one single region is replicated in studies of different centers. The question then rises whether this is related to the low sample size of these studies, the selection of non-representative patient subgroups, or the absence of stratification according to clinical characteristics. Another possibility is that VBM is not a good tool to study functional pathologies such as tinnitus, in contrast to pathologies like Alzheimer’s disease where it is known the pathology is related to cell loss. In a large sample of 154 tinnitus patients VBM and QEEG (Quantitative Electroencephalography) was performed and evaluated by a regression analysis. Correlation analyses are performed between VBM and QEEG data. Uncorrected data demonstrated structural differences in grey matter in hippocampal and cerebellar areas related to tinnitus related distress and tinnitus duration. After control for multiple comparisons, only cerebellar VBM changes remain significantly altered. Electrophysiological differences are related to distress, tinnitus intensity, and tinnitus duration in the subgenual anterior cingulate cortex, dorsal anterior cingulate cortex, hippocampus, and parahippocampus, which confirms previous results. The absence of QEEG-VBM correlations suggest functional changes are not reflected by co-occurring structural changes in tinnitus, and the absence of VBM changes (except for the cerebellum) that survive correct statistical analysis in a large study population suggests that VBM might not be very sensitive for studying tinnitus.

## Introduction

At some point in life most people will experience an unexplainable sound, often described as ringing or roaring, in their ears/head which has no apparent external sound source[1]. This phantom sound is also called tinnitus, and is defined as the perception of a sound in the absence of any external sound source. Tinnitus has been related to listening to loud music [2], sudden sensorineural hearing loss [3], use of medication [4] or other causes. Typically, this sensation is reversible and subsides after a few seconds or sometimes after a few days. Although most incidences of tinnitus are temporary, chronic subjective tinnitus occurs in 10–15% of the general population [5] and severely disrupts quality of life in about 2.4% [6], causing considerable distress involving sleep deprivation [7], depression [8], cognitive problems [9], and work impairment [5].

Analogous to neuropathic pain, tinnitus is related to hyperactivity [1; 10; 11] of the central auditory nervous system. Research on functional imaging also provides evidence that non-auditory brain regions are associated with tinnitus [12] such as the insula [13], anterior cingulate cortex [14; 15], posterior cingulate cortex [15], parahippocampal area [16], and the dorsolateral prefrontal cortex [15; 17; 18; 19]. However, the obtained results of functional neuroimaging are not straightforward as different studies report differences in which non-auditory brain areas are involved.

Apart from functional changes, structural changes in grey matter have been shown to be involved in the pathogenesis of tinnitus [20; 21; 22; 23; 24]. Using voxel-based morphometry (VBM) grey matter changes were shown in tinnitus patients in comparison to control subjects in both the auditory and limbic systems. It was shown that in the thalamus grey matter increases while in the subcallosal frontal cortex grey matter decreases in tinnitus patients [22]. Grey matter decreases have also been shown in the right inferior colliculus and the left hippocampus for tinnitus patients relative to controls [20; 25]. However, grey matter also decreases in individuals with hearing loss without tinnitus in the anterior cingulate and superior medial frontal gyri in comparison to patients with tinnitus and hearing loss [21]. This was further explored by Melcher et al. revealing that the subcallosal brain regions negatively correlate with supra-clinical frequencies (>8 kHz), but not with tinnitus [26]. These findings question the relationship between tinnitus and the structural changes. In addition, it is important to note that most VBM results in tinnitus populations were not obtained using whole brain analysis, but only on supplementary region of interest analyses.

After reviewing both the functional and structural studies together, it is quite surprising that the different studies do not converge to the same results. Low sample size of these studies, the selection of non-representative patient subgroups, or the absence of stratification according to clinical characteristics may explain the lack of agreement between the two studies. Another possibility is that VBM is not an ideal tool to study functional pathologies such as tinnitus, in contrast to pathologies like Alzheimer’s disease where it is known the pathology is related to cell loss [27; 28]. Preliminary evidence by functional imaging studies using QEEG (Quantitative Electroencephalography: i.e. computerized software based analysis of the digitally recorded EEG data, initially limited to Fourier transforms quantitatively expressing the amount of delta, theta, alpha, beta and gamma band activity, but now also extending to Hilbert or wavelet transforms, independent component analyses, source analysis methods etc.) and MEG indicate that depending on the clinical characteristics, the neurophysiological mechanism differs in tinnitus patients. It was demonstrated that depending on the tinnitus lateralization [29], tinnitus sound [30], tinnitus accompanied with distress or not [14; 15], and tinnitus duration [31; 32], different brain areas are involved.

The aim of this study is to verify whether VBM reflects structural changes that might accompany functional changes in tinnitus. This is done by verifying whether (1) Structural (VBM) and electrophysiological changes (QEEG) are correlated, and (2) specific functional and structural changes in the brain of tinnitus patients depend on differences in tinnitus characteristics explaining the variability in previously published data. We opt to use QEEG above fMRI as (1) QEEG measures brain activity more directly, while fMRI indirectly measures using BOLD, (2) fMRI inherently generates noise from the scanner (up to 130 dB) [33] which is problematic when performing research in tinnitus patients, and (3) results reported on tinnitus using fMRI are mainly region of interest analyses and task related, while QEEG measures spontaneous resting state activity. Combining VBM and QEEG has previously been applied in epilepsy research which has shown that combining methods improves the understanding of the pathophysiology of epilepsy: focal discharges may arise from areas of structural abnormality [34; 35]. Therefore, we combine a VBM analysis on MRI data with source localized QEEG in a large group of tinnitus patients.

## Material and Methods

### Patients

One hundred-and-fifty-four tinnitus patients (102 males and 52 females) with a mean age of 50.24 years (Sd = 14.28 years) and a mean tinnitus duration of 5.30 years (Sd = 4.02 years) were selected from the multidisciplinary Tinnitus Research Initiative (TRI) Clinic of the University Hospital of Antwerp, Belgium. Individuals with pulsatile tinnitus, Ménière’s disease, otosclerosis, chronic headache, neurological disorders such as brain tumors, and individuals being treated for mental disorders were not included in the study in order to obtain a more homogeneous sample. In addition, patients with multiple percepts (e.g. perceiving both a pure tone and narrow band noise tinnitus) or with a broadband percept were not included in the study. A combination of a pure tone and noise-like percept is rare and would add an extra condition to the regression analysis. The study was approved by the local ethical committee (Antwerp University Hospital) and was in accordance with the declaration of Helsinki. All patients signed a written informed consent.

All patients were interviewed to determine their perceived location of the tinnitus (unilateral or bilateral), tinnitus duration, and for qualities of their unique tinnitus tone (pure tone like tinnitus or noise-like tinnitus). In addition, all patients were screened for the extent of hearing loss using a pure tone audiometry in accordance with British Society of Audiology procedures at .125 kHz, .25 kHz, .5 kHz, 1 kHz, 2 kHz, 3 kHz, 4 kHz, 6 kHz and 8 kHz [36] (see S1 Fig.). Tinnitus patients were tested for the tinnitus frequency using tinnitus matching analysis. In unilateral tinnitus patients, the tinnitus analysis was performed contralaterally to the tinnitus ear. In bilateral tinnitus patients, tinnitus analysis was performed contralaterally to the worst tinnitus ear. The tinnitus matching analysis consisted of the assessment of the tinnitus pitch and loudness. Depending whether a patient perceives a pure tone or narrow band noise, a 1 kHz pure tone or a narrow band noise around 1kHz (1/3 of an octave above and below the center frequency) was presented contralateral to the (worst) tinnitus ear at 10 dB above the patient’s hearing threshold in that ear. The pitch was adjusted until the patient judged the sound to resemble his/her tinnitus most correctly. The loudness of this tone was then adjusted in a similar way until it corresponded to the patient’s specific tinnitus loudness as well. The tinnitus loudness (dB SL) was computed by subtracting the absolute tinnitus loudness (dB HL) with the auditory threshold at that frequency [37; 38]. Only patients able to successfully perform pitch and loudness matching were included in the study.

A numeric rating scale (NRS) for loudness (‘How loud is your tinnitus?’: 0 = no tinnitus and 10 = as loud as imaginable’) was assessed in addition to the Dutch translation of the Tinnitus Questionnaire validated by Meeus et al. [39] which measures tinnitus related distress. This scale is comprised of 52 items and is a well-established measure for the assessment of a broad spectrum of tinnitus-related psychological complaints. The TQ measures emotional distress, cognitive distress, intrusiveness, auditory perceptual difficulties, sleep disturbances, and somatic complaints. As previously mentioned, the global TQ score can be computed to measure the general level of psychological and psychosomatic distress. In several studies, this measure has been shown to be a reliable and valid instrument in different countries [40; 41]. A 3-point scale is given for all items, ranging from ‘true’ (2 points) to ‘partly true’ (1 point), and ‘not true’ (0 points). The total score (from 0–84) was computed according to standard criteria published in previous work [39; 41; 42]. For the clinical and demographic characterization of the sample see Table 1.

**Table 1:** Clinical and demographic characterization.

Characteristics		
Gender	*Male*	102
	*Female*	52
Age	*Mean*	50.24
	*Sd*	14.28
	*Range*	18–81
Tinnitus Type	*Pure tone*	63
	*Narrow band noise*	87
Tinnitus Lateralization	*Unilateral*	44
	*Bilateral*	110
Tinnitus related distress	*Mean*	36.02
	*Sd*	16.32
	*Range*	2–75
Tinnitus loudness	*Mean*	5.02
	*Sd*	2.48
	*Range*	0–10
Tinnitus duration	*Mean*	5.30
	*Sd*	4.02
	*Range*	1–25
Tinnitus frequency	*Mean*	5104.39
	*Sd*	3166.06
	*Range*	250–12500
Tinntius sensation level	*Mean*	7.76
	*Sd*	8.58
	*Range*	0–40
Mean hearing loss	*Mean*	31.26
	*Sd*	(9.34)
	*Range*	6.3–76

### Data acquisition

1. VBM

Images were acquired using a 3.0 Tesla Siemens Trio scanner. A high resolution scan (MPRAGE) was performed for each subject. TR = 2300 ms, TE = 2.94 ms, inversion time (TI) = 900 ms, flip angle = 9°, 160 sagittal slices, matrix size 256 x 256 mm^2^, 1 x 1 x 1 mm^3^ resolution.

Voxel based morphometry (VBM) is based on high-resolution structural 3D MR images, transformed into a common stereotactic space and is designed to seek significant regional differences by applying voxel-wise statistics in the context of Gaussian random fields [43].

2. QEEG data collection

QEEG recordings (Mitsar-201, NovaTech http://www.novatecheeg.com/) were obtained in a quiet and dimly lit room with each participant sitting upright on a small but comfortable chair. Participants were requested to refrain from alcohol consumption 24 hours prior to recording, and from caffeinated beverages consumption on the day of recording. The actual recording lasted approximately 5 min. The QEEG was sampled with 19 electrodes in the standard 10–20 International placement referenced to linked ears and impedances were checked to remain below 5 kΩ. Data was collected with the patient’s eyes-closed (sampling rate = 1024Hz, band passed 0.15–200Hz). This method is already applied in previous research [15; 32].

### Data processing

1. VBM

Statistical parametric software (SPM8, Welcome Trust Center for Neuroimaging, http://www.fil.ion.ucl.ac.uk/spm/software/spm8) was used to analyze the data. Image preprocessing was performed following an optimized-VBM-protocol by using VBM8 (http://dbm.neuro.uni-jena.de/vbm.htlm) with default parameters. All images were partitioned into grey and white matter and cerebrospinal fluid (CSF). Images were bias-corrected, tissue classified, and registered using linear (12-parameter affine) and non-linear transformations (warping), within a unified model [44]. Subsequently, analyses were performed on the density of the gray matter (GM) segments, which were multiplied by the non-linear components derived from the normalization matrix in order to preserve actual GM and WM values locally (modulated GM and WM volumes). The resulting grey matter images were smoothed with a Gaussian kernel of 8 mm full width at half maximum (FWHM).

For the region of interest analysis, we used a mask of cortical and subcorticortical regions that were obtained with QEEG. In order to maintain compatibility of the results obtained with QEEG and MRI, we used the corresponding Brodmann areas as defined in Montreal-Neurological-Institute (MNI)-coordinate space with the WFU-Pickatlas [45].

Anatomical labeling of significant clusters was done by means of the anatomical automatic labeling toolbox (AAL) [46].

2. QEEG & Source Localization

Data was resampled to 128 Hz, band-pass filtered (fast Fourier transform filter) to 2–44 Hz, and subsequently transposed into Eureka! Software [47] where it was plotted and carefully inspected for artifacts (i.e. eye blinks, eye movements, teeth clenching, body movement, or ECG artifact) which were removed. Using a fast Fourier transform for the different frequency bands, the power for the delta (2–3.5 Hz), theta (4–7.5 Hz), alpha (8–12Hz), beta (13–30 Hz) and gamma (30.5–44 Hz) were computed.

Standardized low-resolution brain electromagnetic tomography (sLORETA) was used to estimate the intracerebral electrical sources that generated the scalp-recorded activity in each of the five frequency bands [48]. sLORETA computes electric neuronal activity as current density (A/m^²^) without assuming a predefined number of active sources. The sLORETA solution space consists of 6,239 voxels (voxel size: 5 x 5 x 5 mm). Computations were made in a realistic head model, and were restricted to cortical grey matter and hippocampi, as defined by digitized MNI152 template [49]. Scalp electrode coordinates on the MNI brain are derived from the international 5% system [50]. Thus, sLORETA images represent the standardized electric activity at each voxel in neuroanatomic Montreal Neurological Institute (MNI) space as the exact magnitude of the estimated current density. Anatomical labels as Brodmann areas are also reported using MNI space, with correction to Talairach space [51].

In addition, the log-transformed electrical current density was averaged across all voxels belonging to the region of interest that were obtained for the whole-brain analysis for the specific frequency band.

### Statistical analyses

1. VBM

For the statistical analysis, we excluded all voxels with a grey matter value below 0.1 (maximum value: 1) to avoid possible edge effects around the border between grey and white matter, and to include only voxels with sufficient grey matter proportion. A whole brain multiple regression analysis was conducted using age, gender, tinnitus type, tinnitus lateralization, tinnitus related distress, tinnitus loudness, tinnitus duration, tinnitus frequency, and tinnitus sensation level as independent variables to test for any association with changes in grey matter. These independent variables were selected based on previous research which demonstrated that these different characteristics have an influence on brain activity or connectivity in tinnitus; namely age [52], gender [53], tinnitus lateralization [16; 17], tinnitus sound type [30], tinnitus loudness [54], distress [15; 55], tinnitus duration [31; 32], frequency and sensation level [21]. We included both the tinnitus loudness as well as tinnitus sensation level, because the tinnitus loudness is a subjective measure of how loud the patient perceives the tinnitus, and the tinnitus sensation level is a more objective measure of the tinnitus including a control for hearing loss at the tinnitus frequency. The tinnitus loudness matching in dB sensation level is calculated by subtracting the hearing loss in dB from the intensity of the presented sound in dB HL in order to compensate for hearing loss at the tinnitus frequency. In view of the fact that the tinnitus normally matches the area of hearing loss [56], this implies that hearing loss is controlled for. Both measures in our population do not correlate (r = .06, *n*.*s*.).

In addition to the whole brain analysis, we performed a region of interest analysis using a mask of cortical and subcortical regions in the auditory system previously used [20; 21]. We used the same anatomical mask wherein the region of interest encompassed the ventral and dorsal cochlear nuclei (sphere radius, 5 mm; Montreal-Neurological-Institute (MNI)-coordinates, ±10, −38, −45), superior olivary complex (sphere radius, 5 mm; MNI-coordinates, ±13, −35, −41), inferior colliculus (sphere radius, 5 mm; MNI-coordinates, ±6, −33, −11), medial geniculate nucleus (sphere radius, 8 mm; MNI coordinates, ±17, −24, −2), and the primary and secondary auditory cortices corresponding to Brodmann areas 41, 42, and 22 (defined with the WFU-Pick Atlas [45]). The main advantage of using *a priori* region of interest testing over whole-brain analysis is that it limits type I errors by reducing the number of statistical tests to only a few regions of interest. Again, a multiple regression analysis was conducted with age, gender, tinnitus type, tinnitus lateralization, tinnitus related distress, tinnitus loudness, tinnitus duration, tinnitus frequency, and tinnitus sensation level as independent variables to test for any association with changes in grey matter within the regions of interest.

To verify the obtained VBM results we applied a supplementary analysis for which we assigned each subject randomly to one of two smaller groups and conducted a similar analysis (split-half analysis).

In addition, we also performed three simple regression analyses on respectively distress, duration and the tinnitus loudness as separate independent variables and test for any association with changes in grey matter. We selected these three variables as previous research has shown that these parameters play an important role in tinnitus [57]. It is important to verify whether similar results can be obtained for different tinnitus characteristics separately. To some extent, results within the integrative model can be biased due to intercorrelations between the different tinnitus characteristics. Therefore, we conducted additional analyses including only one regressor each time (tinnitus distress, loudness, and duration).

In addition to the previously mentioned analyses we also applied a simple regression on the hearing loss to further confirm previous research findings on hearing loss and verify whether hearing loss does have a great impact on grey matter. In addition we applied a multiple regression analysis with hearing loss, tinnitus loudness, tinnitus distress, and tinnitus duration as independent variables to compare what the influence is from respectively hearing loss and the tinnitus characteristics (loudness distress and duration).

For all the analyses the statistical significance was set at *p* < .001 uncorrected and *p* < .05 false discovery rate (FDR) corrected at voxel–level and the minimum contiguous cluster size at 20 voxels, except when the effect was strong, the cluster size was set at 50 voxels (i.e. for hearing loss)

2. Source localization

sLORETA was used to perform a voxel-by-voxel analysis (comprising 6239 voxels) for the different frequency bands to regress of the current density distribution to identify potential differences in brain electrical activity. Nonparametric statistical analyses of functional sLORETA images (statistical nonparametric mapping: SnPM) were performed for each contrast using sLORETA’s built-in voxel wise randomization tests (5000 permutations) and employing a *F*-statistic with a threshold *P* < 0.05. As explained by Nichols and Holmes, the statistical nonparametric mapping method does not rely on an assumption of a Gaussian distribution for the validity and corrects for all multiple comparisons (i.e. for the collection of test performed for all voxels and for all frequency bands) by employing a locally pooled (smoothed) variance estimate the outperforms of the comparable statistical parametric mapping [58; 59]. A multiple regression analysis was conducted with age, gender, tinnitus type, tinnitus lateralization, tinnitus related distress, tinnitus loudness, tinnitus duration, tinnitus frequency, tinnitus sensation level and hearing loss as independent variables. We also applied a simple regression on the hearing loss to further confirm previous research findings on hearing loss the QEEG analysis.

3. The combination of source localized QEEG and VBM

A whole brain analysis correlating activity of QEEG and VBM was conducted, as well as region of interest analysis on the regions obtained with source localized QEEG to increase spatial accuracy. That is the dorsal and subgenual anterior cingulate cortex, the parahippocampus and auditory cortex for specific frequency obtained. These regions of interest were then correlated using a Pearson correlation to the same region of interest on the VBM data. Thalamic and cerebellum region of interests were not included in this subsection as this is not possible to locate with source localized QEEG.

## Results

### VBM

1. Integrative model: Tinnitus

**Fig 1:**
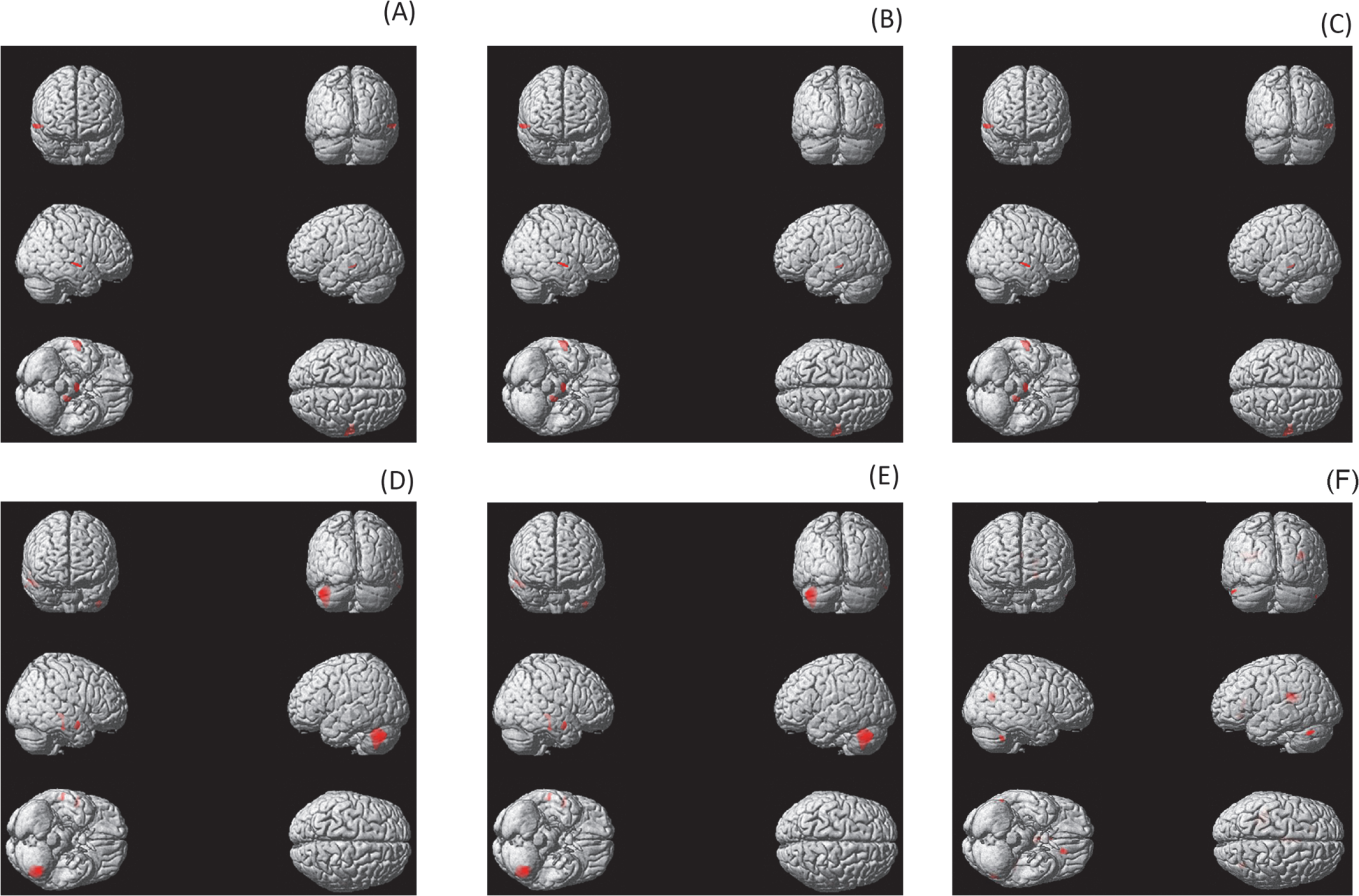
Grey matter concentration for (A) for tinnitus lateralization. Grey matter decrease projected onto a T1-weighted image. Maximum intensity projection with a threshold of *p* < .001 (uncorrected) at both voxel and cluster level. Coordinates of peak voxel (see Table 2 & S2 Table). (B) Grey matter concentration increases for tinnitus distress. Grey matter increase projected onto a T1-weighted image. Maximum intensity projection with a threshold of *p* < .001 (uncorrected) at both voxel and cluster level. Coordinates of peak voxel (see Table 2 & S2 Table). (C) Grey matter concentration decreases for tinnitus distress. Grey matter decrease projected onto a T1-weighted image. Maximum intensity projection with a threshold of *p* < .001 (uncorrected) at both voxel and cluster level. Coordinates of peak voxel (see Table 2 & S2 Table). (D) Grey matter concentration decreases for tinnitus loudness Grey matter decrease projected onto a T1-weighted image. Maximum intensity projection with a threshold of *p* < .001 (uncorrected) at both voxel and cluster level. Coordinates of peak voxel (see Table 2 & S2 Table). (E) Grey matter concentration decreases for tinnitus distress. Grey matter decrease projected onto a T1-weighted image. Maximum intensity projection with a threshold of *p* < .001 (uncorrected) at both voxel and cluster level. Coordinates of peak voxel (see Table 2 & S2 Table). (F) Grey matter concentration decreases for hearing loss. Grey matter decrease projected onto a T1-weighted image. Maximum intensity projection with a threshold of *p* < .001 (uncorrected) at both voxel and cluster level. Coordinates of peak voxel (see Table 3 & S3 Table).

The whole brain analysis showed only a significant effect for the grey matter density changes after correction at voxel–level for tinnitus distress (Z = 4.29, *p*_*corrected*_ = .02), tinnitus loudness (Z = 4.22, *p*_*corrected*_ = .03), and tinnitus duration (Z = 4.25, *p*_*corrected*_ = .04) (see Table 2 for overview). These results demonstrated that the higher patient’s score on tinnitus distress, tinnitus loudness, and tinnitus duration, the more grey matter density was decreased (see Fig. 1; uncorrected results see S1 Text & S1 Table).

**Table 2:** Local maxima from the different contrasts highlighting grey matter differences for tinnitus type, tinnitus lateralization, TQ, Vas loudness, Tinnitus duration, Tinnitus frequency and Tinnitus sensation level (N = 154).

			Coordinates			Significance		Score		Cluster Size
			MNI			*p* FDR corrected at voxel level	*p* uncorrected	Z		Voxels
			x	y	z					
*1. Age*										
+										
	n.r.o.									
-										
	n.r.o.									
*2. Gender*										
+										
	n.r.o.									
-										
	n.r.o.									
*3. Type (NBN vs. PT)*										
+										
	n.r.o.									
-										
	n.r.o.									
*4. Lateralization (Unilateral vs. Bilateral)*										
+										
	n.r.o.									
-										
	n.r.o.									
*5. TQ (tinnitus related distress)*										
+										
	n.r.o.									
-										
	Cerebellum VIIb	L	-32	-36	-39	.02	< .001		4.29	464
			-38	-55	-45					
			-50	-73	-30					
*6.Tinnitus loudness*										
+										
	n.r.o.									
-										
	Crus I	L	-62	-52	-26	.03	< .001		4.22	68
*7. Duration*										
+										
	n.r.o.									
-										
	Cerebellum X	R	21	-37	-41	.04	< .001		4.25	703
			29	-36	-45					
*8. Tinnitus Frequency*										
+										
	n.r.o.									
-										
	n.r.o.									
*9. Tinnitus Sensation Level*										
+										
	n.r.o.									
-										
	n.r.o.									

n.r.o. = no results obtained; R: right; L: left.

To verify the VBM results we conducted an additional analysis for which we assigned each subject randomly to one of two smaller groups and conducted a similar analysis (see S2 Table). Again our results demonstrated that the higher the patient’s score on tinnitus distress, tinnitus loudness and tinnitus duration, the more grey matter density was decreased mainly in the cerebellum. However, these findings are only present when they remain uncorrected. After correction no significant results were obtained.

We also conducted a region of interest analysis for the entire auditory system. We detected a smaller grey matter density for the unilateral tinnitus patients in comparison to the bilateral tinnitus patients in the right primary auditory cortex, which is in association with tinnitus lateralization (Z = 2.86, *p* = .002 uncorrected). However, no other changes were obtained in respectively the ventral and dorsal cochlear nuclei, inferior colliculus, medial geniculate nucleus as well as the primary and secondary auditory cortices in relation to age, gender, tinnitus type, tinnitus lateralization, tinnitus related distress, tinnitus loudness, tinnitus duration, tinnitus frequency and tinnitus sensation level.

2. Integrative model: Tinnitus and hearing loss

The whole brain analysis revealed a significant effect for the grey matter density changes after correction at voxel–level for hearing, (see Table 3 for overview). These results demonstrated that the more hearing loss patients have the more grey matter density was decreased in the auditory cortex (Z = 4.86, *p*_*corrected*_ = .007) and thalamus (Z = 4.77, *p*_*corrected*_ = .02). No effects were obtained for the tinnitus characteristics (see Fig. 1, uncorrected results see S2 Text & S3 Table).

**Table 3:** Local maxima from the different contrasts highlighting grey matter differences for tinnitus type, tinnitus lateralization, TQ, Vas loudness, Tinnitus duration, Tinnitus frequency and Tinnitus sensation level and hearing loss (N = 154).

			Coordinates			Significance		Score	Cluster Size
			MNI			*p* FDR corrected at voxel level	*p* uncorrected	Z	Voxels
			x	y	z				
*1. Age*									
+									
	n.r.o.								
-									
	n.r.o.								
*2. Gender*									
+									
	n.r.o.								
-									
	n.r.o.								
*3. Type (NBN vs. PT)*									
+									
	n.r.o.								
-									
	n.r.o.								
*4. Lateralization (Unilateral vs. Bilateral)*									
+									
	n.r.o.								
-									
	n.r.o.								
*5. TQ (tinnitus related distress)*									
+									
	n.r.o.								
-									
	n.r.o.								
*6.Tinnitus loudness*									
+									
	n.r.o.								
-									
	n.r.o.								
*7. Duration*									
+									
	n.r.o.								
-									
	n.r.o.								
*8. Tinnitus Frequency*									
+									
	n.r.o.								
-									
	n.r.o.								
*9. Tinnitus Sensation Level*									
+									
	n.r.o.								
-									
	n.r.o.								
*10. Hearing loss*									
+									
	n.r.o.								
-									
	Auditory Cortex	L	-29	-36	19	.007	< .001	4.86	1850
	Thalamus	L	0	-6	21	.02	< .001	4.77	808

n.r.o. = no results obtained; R: right; L: left.

Since the different variables included in the integrative model are to some extent intercorrelated which could bias the results, we conducted an additional analysis including only one regressor. We applied this method with tinnitus distress, tinnitus duration and tinnitus loudness as regressors on the whole brain. We also applied a similar method for hearing loss as previous research already indicated that hearing loss might be a more important variable to explain the grey matter density in tinnitus patients.

3. Single regression model: Tinnitus Distress

**Fig 2:**
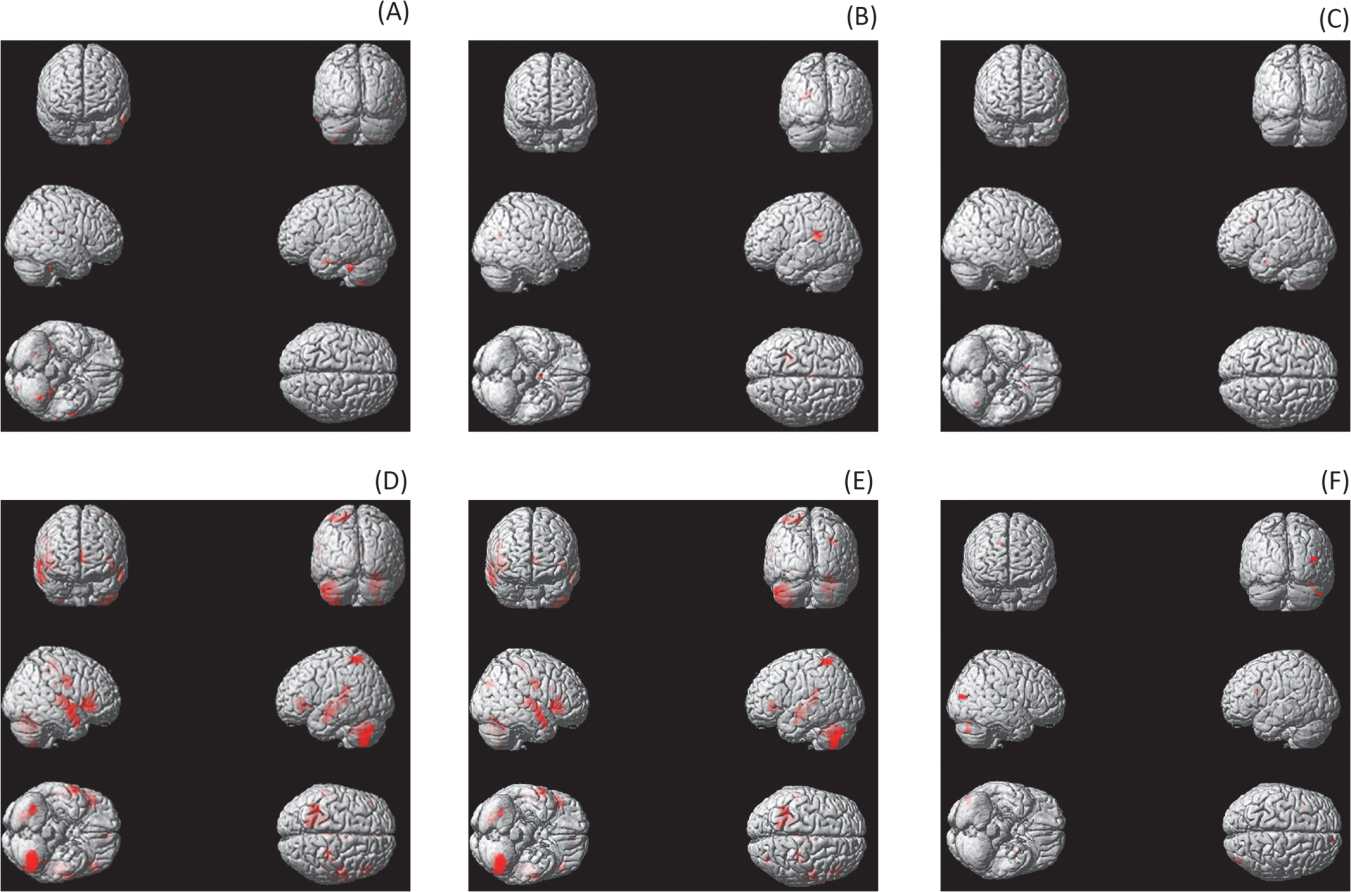
Grey matter concentration decreases for (A) tinnitus duration. Grey matter decrease projected onto a T1-weighted image. Maximum intensity projection with a threshold of *p* < .001 (uncorrected) at both voxel and cluster level. Coordinates of peak voxel (see S2 Table). (B) Grey matter concentration decreases for NRS loudness. Grey matter decrease projected onto a T1-weighted image. Maximum intensity projection with a threshold of *p* < .001 (uncorrected) at both voxel and cluster level. Coordinates of peak voxel (see S3 Table). (C) Grey matter concentration decreases for tinnitus duration. Grey matter decrease projected onto a T1-weighted image. Maximum intensity projection with a threshold of *p* < .001 (uncorrected) at both voxel and cluster level. Coordinates of peak voxel (see S4 Table). (D) Grey matter concentration decreases for hearing loss. Grey matter decrease projected onto a T1-weighted image. Maximum intensity projection with a threshold of *p* < .05 (corrected) at both voxel and cluster level. Coordinates of peak voxel (see Table 4). (E) Grey matter concentration decreases for hearing loss controlling for tinnitus characteristics (distress, loudness and duration). Grey matter decrease projected onto a T1-weighted image. Maximum intensity projection with a threshold of *p* < .05 (corrected) at both voxel and cluster level. Coordinates of peak voxel (see S5 Table). (F) Grey matter concentration decreases for tinnitus characteristics controlling for hearing loss. Grey matter decrease projected onto a T1-weighted image. Maximum intensity projection with a threshold of *p* < .001 (uncorrected) at both voxel and cluster level. Coordinates of peak voxel (see S6 Table).

When applying a whole brain analysis with tinnitus distress as the single regressor, a significant effect for the cerebellar activity (Z = 5.48, *p*_*corrected*_ < .001) after correction at voxel–level, again reveals a decrease in grey matter density analogous with more distress (see Table 4; see Fig. 2A). However, several other areas where also shown to have a decrease in grey matter in correlation with more distress such as in the cerebellum, the dorsal lateral prefrontal cortex, the posterior cingulate cortex, the inferior temporal cortex, and the posterior middle temporal cortex. However, these findings were significantly uncorrected.

**Table 4:** Local maxima from the different contrasts highlighting grey matter differences for TQ (N = 154).

			Coordinates			Significance		Score	Cluster Size
			MNI			*p* FDR corrected at voxel level	*p* uncorrected	Z	Voxels
			x	y	z				
+									
	n.r.o.								
-									
	Cerebellum VIII	L	-30	-36	-38	.01	< .001	5.48	497
		L	-24	-35	-30	.18	< .001	4.96	
		L	-35	-42	-35	.32	< .001	4.63	
	Cerebellum VIII	R	29	-51	-50	.18	< .001	5.03	20
	Cerebellum V	L	-39	-54	-56	.18	< .001	5.03	133
		L	-33	-60	-62	.95	< .001	3.82	
		L	-30	-62	-53	.96	< .001	3.40	
	Posterior cingulate cortex	L	2	-39	26	.23	< .001	4.80	42
	Cerebellum VI	R	39	-41	-35	.39	< .001	4.56	106
		R	32	-38	-33	.52	< .001	4.29	
		R	35	-48	-27	.96	< .001	3.56	
	Inferior temporal cortex	L	-65	-27	-26	.40	< .001	4.50	22
	Dorsal anterior cingulate cortex	L	2	35	27	.40	< .001	4.49	23
	Temporal parietal junction	R	66	-26	12	.43	< .001	4.39	21
	Anterior inferior temporal cortex		-62	-9	-29	.43	< .001	4.38	119
			-57	6	-29	.48	< .001	4.32	
			-56	0	-23	.88	< .001	3.94	
	Cerebellum X	R	-17	-71	26	.62	< .001	4.17	20
		L	26	-66	-62	.76	< .001	4.01	20
		R	-24	-87	-41	.92	< .001	3.88	23
	Posterior middle temporal cortex	R	60	-50	4	.95	< .001	3.84	30
			59	-59	4	.96	< .001	3.39	
	Cerebellum IX	R	24	-54	-59	.96	< .001	3.69	31
		R	18	-59	-56	.96	< .001	3.58	

n.r.o. = no results obtained; R: right; L: left.

4. Single regression model: Tinnitus Duration

When applying a whole brain analysis with duration as the single regressor, no significant effect could be obtained after correction at voxel–level (see Table 5; see Fig. 2B). However, uncorrected demonstrates a significant effect in the right parahippocampus and the superior temporal pole areas revealing a decrease in grey matter in relationship with tinnitus duration.

**Table 5:** Local maxima from the different contrasts highlighting grey matter differences for duration (N = 154).

			Coordinates			Significance		Score	Cluster Size
			MNI			*p* FDR corrected at voxel level	*p* uncorrected	Z	Voxels
			x	y	z				
+									
	n.r.o.								
-									
	Parahippocampus	R	32	-18	-30	.76	< .001	3.25	24
	Superior Temporal Pole	R	47	18	-30	.76	< .001	3.24	180

n.r.o. = no results obtained; R: right; L: left.

5. Single regression model: Tinnitus Loudness

A whole brain analysis with tinnitus loudness as the single regressor indicated no significant effect could be obtained after correction at voxel–level (see Table 6; see Fig. 2C). However, uncorrected demonstrates a significant effect in the dorsal lateral prefrontal cortex, caudate nucleus, inferior temporal cortex, cerebellum, and putamen revealing a decrease in grey matter in relationship with an increase in tinnitus loudness.

**Table 6:** Local maxima from the different contrasts highlighting grey matter differences for loudness (N = 154).

			Coordinates			Significance		Score	Cluster Size
			MNI			*p* FDR corrected at voxel level	*p* uncorrected	Z	Voxels
			x	y	z				
+									
	n.r.o.								
-									
	Dorsal Lateral Prefrontal Cortex	L	-48	20	38	.67	< .001	4.99	28
	Caudate nucleus	L	-14	20	-9	1.00	< .001	4.35	79
			-14	15	0	1.00	< .001	3.47	
	Inferior temporal cortex	L	-60	0	-23	1.00	< .001	4.11	28
	Dorsal Lateral Prefrontal Cortex	L	-20	35	32	1.00	< .001	3.97	33
	Cerebellum VIII	L	-32	-44	-29	1.00	< .001	3.91	30
		L	-23	-51	-26	1.00	< .001	3.73	
	Cerebellum VI	L	-42	-57	-56	1.00	< .001	3.66	23
		L	-35	-53	-56	1.00	< .001	3.41	
	Caudate nucleus	R	15	24	-2	1.00	< .001	3.64	155
		R	11	14	-5	1.00	< .001	3.61	
		R	15	17	3	1.00	< .001	3.54	
	Caudate nucleus	L	-14	24	7	1.00	< .001	3.61	20
	Putamen	L	-21	14	0	1.00	< .001	3.52	25
		L	-27	8	-3	1.00	.001	3.25	

n.r.o. = no results obtained; R: right; L: left.

6. Single regression model: Hearing Loss

When applying a whole brain analysis with hearing loss as the single regressor, several significant effects were obtained after correction at voxel–level, revealing a decrease in grey matter density associated with an increase in hearing loss (see Table 7; see Fig. 2D). Decrease in grey matter was obtained in the cerebellum, the ventral lateral prefrontal cortex, the somatosensory cortex, the auditory cortex, the posterior cingulate cortex, and the superior parietal cortex.

**Table 7:** Local maxima from the different contrasts highlighting grey matter differences for hearing loss controlling for the tinnitus characteristics (Distress, Loudness and Duration) (N = 154).

			Coordinates			Significance	Score	Cluster Size
			MNI			FDR corrected at voxel level	Z	Voxels
			x	y	z			
+								
	n.r.o.							
-								
	Cerebellum Crus I	L	-39	-62	-39	< .001	7.14	8055
		L	-48	-60	-65	.02	5.83	
	Ventral lateral prefrontal cortex	R	45	24	-3	.01	6.26	8944
		R	57	-14	7	.02	5.83	
		R	47	29	9	.03	5.74	
	Ventral lateral prefrontal cortex	L	-41	32	4	.01	6.21	1226
	Somatosensory cortex	R	51	-11	32	.01	6.13	3118
		R	30	-27	57	.02	5.83	
		R	45	-39	47	.24	5.05	
	Auditory cortex	L	-50	-30	6	.02	5.88	4905
		L	-45	-9	-9	.03	5.69	
		L	-57	-14	-14	.06	5.49	
	Posterior cingulate cortex	R	2	-30	44	.02	5.85	1535
		R	9	-9	47	.06	5.44	
		L	-5	5	44	.41	4.88	
	Superior Parietal cortex	L	-26	-50	60	.02	5.80	2937
		L	-36	-50	62	.04	5.61	
		L	-23	-35	62	.21	5.09	
	Cerebellum Crus I	R	23	-72	-38	.04	5.58	5622
		R	30	-74	-17	.05	5.48	
		R	29	-53	-14	.08	5.38	

n.r.o. = no results obtained; R: right; L: left.

7. A comparison between hearing loss and tinnitus characteristics (Distress, Loudness and Duration).

A whole brain analysis including in the contrast hearing loss and the tinnitus characteristics as covariates revealed a significant effect after correction at voxel-level, demonstrating a decrease in grey matter density (see Table 8; Fig. 2E). Decrease in grey matter was obtained in the cerebellum (Z = 7.14, *p*_*corrected*_ < .001), ventral lateral prefrontal cortex (Z = 6.26, *p*_*corrected*_ = .01), somatosensory cortex (Z = 6.13, *p*_*corrected*_ = .01), auditory cortex (Z = 5.88, *p*_*corrected*_ = .02), posterior cingulate cortex (Z = 5.85, *p*_*corrected*_ = .02), superior parietal cortex (Z = 5.61, *p*_*corrected*_ = .05), and crus I (Z = 5.48, *p*_*corrected*_ = .05).

**Table 8:** Local maxima from the different contrasts highlighting grey matter differences for tinnitus characteristics (Distress, Loudness and Duration) controlling for hearing loss (N = 154).

			Coordinates			Significance		Score	Cluster Size
			MNI			*p* FDR corrected at voxel level	*p* uncorrected	Z	Voxels
			x	y	z				
-									
	Middle temporal cortex	R	39	-82	13	.70	<.001	3.29	365
	Anglur gyrus/ Precuneus	L	51	-87	102	.70	<.001	3.19	1584
		L	36	-81	101	.70	<.001	2.88	
	Insula	L	-30	18	21	.70	<.001	3.04	376
	Cerebellum Crus I	R	71	-114	-50	.70	<.001	3.01	2099

n.r.o. = no results obtained; R: right; L: left.

A similar analysis including in the contrast of the different tinnitus characteristics and hearing loss as a covariates revealed a significant effect uncorrected, however after correction the effect disappeared (see Table 9; Fig. 2F). Decrease in grey matter was obtained in the cerebellum (Z = 3.01 *p*_*uncorrected*_ < .001), angular gyrus (Z = 3.19 *p*_*uncorrected*_ < .001), insula (Z = 2.88 *p*_*uncorrected*_ < .001) and middle temporal cortex (Z = 3.29 *p*_*uncorrected*_ < .001).

**Table 9:** Local maxima from the different contrasts highlighting activity differences for tinnitus type, tinnitus lateralization, TQ, Vas loudness and duration (N = 154) for the source localized QEEGs.

	Coordinates				Voxel Value
	MNI				
		**X**	**Y**	**Z**	
*1. Age*					
+					
n.r.o.					
-					
n.r.o.					
*2. Gender*					
+					
n.r.o.					
-					
n.r.o.					
*3. Type (NBN vs. PT)*					
+					
n.r.o.					
-					
n.r.o.					
*4. Lateralization (Unilateral vs. Bilateral)*					
+					
n.r.o.					
-					
n.r.o.					
*5. TQ (tinnitus related distress)*					
+					
*Alpha*					
Subgenual anterior cingulate cortex	L/R	0	10	-5	.41
Dorsal anterior cingulate cortex	L/R	5	25	15	.32
Hippocampus	L/R	-10	5	-15	.30
*Beta*					
Dorsal anterior cingulate cortex	L	-10	20	25	.45
n.r.o.					
*6. Tinnitus loudness*					
+					
*Gamma*					
Hippocampus	L/R	-10	35	0	.43
-					
n.r.o.					
*7. Duration*					
+					
*Theta*					
Dorsal anterior cingulate cortex	L	-5	0	40	.42
	R	5	0	45	.39
-					
n.r.o.					
*8. Tinnitus Frequency*					
+					
n.r.o.					
-					
n.r.o.					
*9. Tinnitus Sensation Level*					
+					
n.r.o.					
-					
n.r.o.					
*10*. Hearing loss					
+					
n.r.o.					
-					
n.r.o.					

n.r.o. = no results obtained; R: right; L: left.

### Source localized QEEG

**Fig 3:**
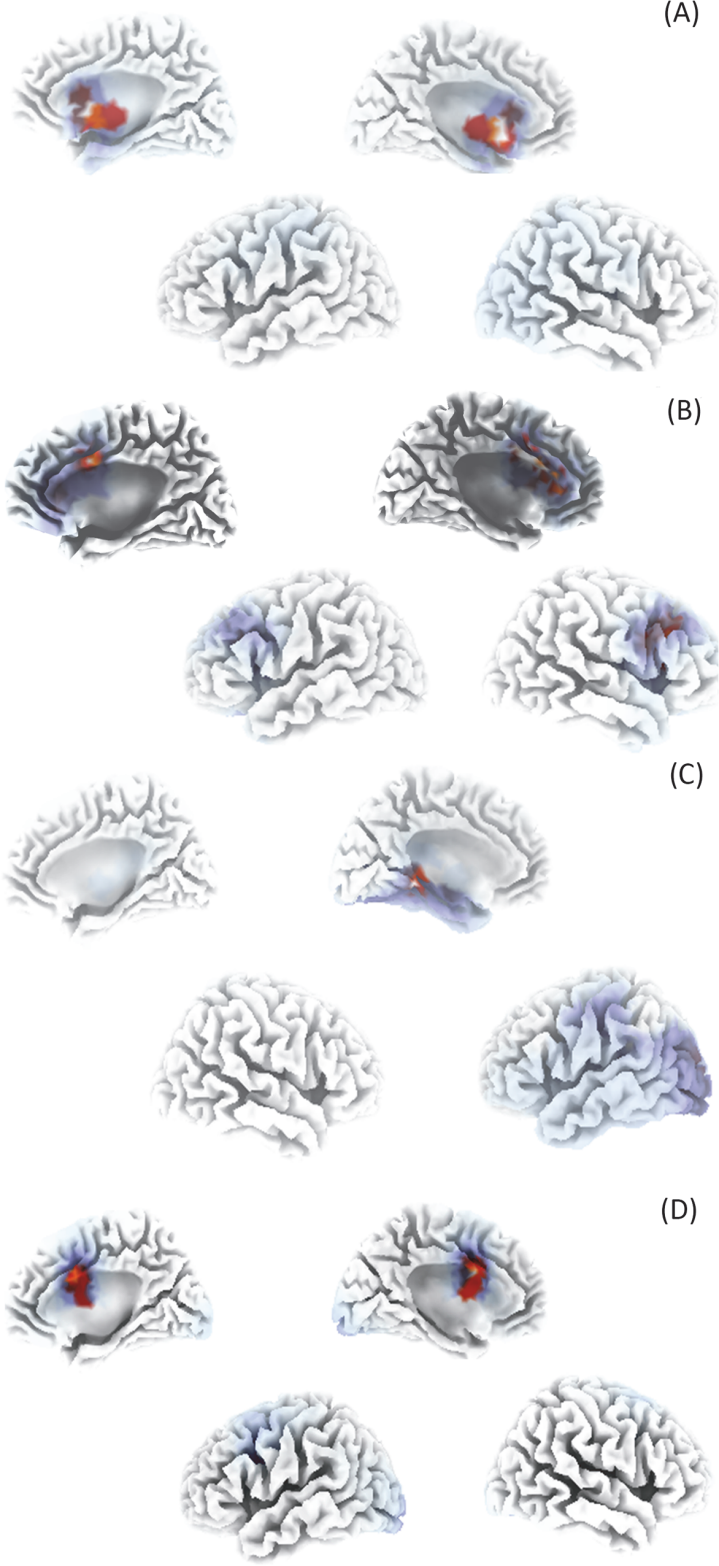
Significant results for current density amplitude analysis in the (A) alpha frequency band. sLORETA current source density in the alpha band correlated positively with tinnitus distress in the subgenual anterior cingulate, dorsal anterior cingulate and the hippocampus. (B) Significant results for current density amplitude analysis in the beta frequency band.in the beta in the dorsal anterior cingulate cortex. This image shows significant results only. (C) Significant results for current density amplitude analysis in the gamma frequency band. sLORETA current source density in the gamma band correlated positively with tinnitus loudness in the left parahippocampal area. This image shows significant results only. (D) Significant results for current density amplitude analysis in the theta frequency band. sLORETA current source density in the theta band correlated positively with tinnitus duration in the dorsal anterior cingulate cortex area This image shows significant results only.

A regressions analysis revealed a significant effect (*p* < .05) for tinnitus related distress, tinnitus loudness and tinnitus duration (See Table 10). A closer look shows a positive relation between tinnitus related distress and respectively the subgenual anterior cingulate cortex, the dorsal anterior cingulate cortex and the hippocampus for the alpha frequency as well as between tinnitus related distress and the hippocampus for the beta frequency (see Fig. 3A-B). Tinnitus loudness has a positive relationship with the left parahippocamal area in the gamma frequency (see Fig. 3C). In addition, tinnitus duration is positively related to increased theta activity within the dorsal anterior cingulate cortex (see Fig. 3D). In addition we applied a single regression analysis to verify whether we could observe a similar result for tinnitus duration (see S2 Fig.), tinnitus related distress (see S3 Fig.), and tinnitus loudness (see S4 Fig.). A simple regression analysis including hearing loss did not yield a significant result.

**Table 10:** Pearson correlation between the region of interest on the sturctural (VBM) and functional (source localized EEG).

	**Frequency Bands**				
	Delta	Theta	Alpha	Beta	Gamma
Subgenual anterior cingulate cortex	.09	.04	.02	.12	.05
Left Dorsal Anterior cingulate cortex	.04	-.02	.03	.05	.04
Right Dorsal Anterior cingulate cortex	-.02	-.06	.03	.09	.10
Left Hippocampus	.10	.03	.08	.06	.05
Right Hippocampus	.05	.04	.07	.04	.06
Left Parahippocampus	-.04	-.04	.04	.05	.06
Right Parahippocampus	-.04	-.03	.09	-.03	-.01
Left Auditory Cortex	-.03	-.02	.05	.09	.10
Right Auditory Cortex	.08	.07	.08	.04	.04

### The combination of source localized QEEG and VBM

A whole brain analysis between the structural data obtained by MRI and functional data obtained by QEEG yielded no significant effect. In addition, no significant effects were obtained between the regions of interest on source localized QEEGs and the region of interest on the VBM data (r = between -06 and .12) (see Table 10).

## Discussion

The aim of this study is to verify whether VBM reflects structural changes that might accompany functional changes in tinnitus. However no correlations could be found between functional and structural changes. In epilepsy it has been shown that focal discharges occur at the areas of structural brain changes, suggesting these structural changes might be causally related to the epilepsy. However in tinnitus, even though structural changes have been described, they do not seem to be related to the oscillatory changes that have been linked to tinnitus. One explanation could be that the structural changes seen in tinnitus relate more to hearing loss, which frequently accompanies tinnitus, than to the tinnitus per se. Another explanation could be that structural changes induce oscillatory changes at a distance, change synchrony or modify connectivity rather than activity.

From a methodological point of view EEG and VBM have nothing in common. Source analyzed EEG can record and correctly localize cortical activity, although it excludes activity from deep nuclei, the brainstem, and/or the cerebellum, whereas VBM is anatomically unrestricted. Although the neural elements in EEG are relatively well defined, it is unclear what the anatomical basis for VBM actually is. VBM likely evaluates structural changes, EEG records functional changes. So, although both techniques could be complementary, this study did not find any correlation between the results of the two used techniques. This could also be related to the fact that they measure unrelated properties of tinnitus. This raises the question whether the VBM technique is ideally suited to study pathologies related to functional changes like tinnitus. No studies have been able to replicate other centers’ results. One option for the variability is selection of the patients, which can bias the obtained results as it has been shown that many brain areas are involved in tinnitus, depending on the investigated tinnitus characteristic.

This study detected structural differences in grey matter within auditory, hippocampal, thalamic, and cerebellar areas that are related to specific tinnitus characteristics. However, these effects were mainly uncorrected. In contrast, significant results were obtained for hearing loss in the cerebellum, ventral lateral prefrontal cortex, somatosensory cortex, auditory cortex, thalamus, posterior cingulate cortex, and superior parietal cortex. These latter results remained after correction for multiple comparisons. In an integrative model including all tinnitus characteristics and hearing loss only an effect for hearing loss was found, demonstrating a decrease in grey matter density in the auditory cortex and the thalamus. These findings corroborate previous research indicating that grey matter decreases can be explained by the hearing loss and not by the tinnitus [21; 26]. Functional (QEEG) differences were obtained for tinnitus distress, tinnitus intensity, and tinnitus duration in the subgenual and dorsal anterior cingulate cortex, hippocampus and the parahippocampus. This is consistent with previously published data [14; 15], suggesting this population is representative and comparable with previously studied populations.

One structural finding that was consistently present, after correction for multiple comparisons, was the changes within the cerebellum. However this effect did not remain if we included hearing loss in the model. That is, tinnitus distress, tinnitus loudness and tinnitus duration are negatively correlated with grey matter within the cerebellum. This was further confirmed by a split-half reliability analysis that again revealed that the higher the patient scored on tinnitus distress, tinnitus loudness, and tinnitus duration the more grey matter density was decreased mainly in the cerebellum. The cerebellum has previously been associated with higher-order functions [60; 61], as well as auditory sensory processing [62]. Cerebellar involvement in auditory processing could be related to auditory prediction, i.e. the cerebellum detects relevant changes in auditory events, such as sound onset, and predicts when the next event is going to occur [63]. It is strange that there are no direct functional connections between the primary auditory cortex and the cerebellum [64], but animal research in cats demonstrated anatomical connections between the cochlear nuclei and parts of the cerebellum that might be the anatomical basis for auditory sensory input to the cerebellum [65]. Recently, it was shown in animals that the paraflocculus of the cerebellum also plays a role in tinnitus [66]. In humans, functional cerebellar anomalies in conjunction with self-reported tinnitus has been documented [67], and PET studies have shown cerebellar activation in gaze evoked tinnitus [68] as well as right cerebellar activation during tinnitus perception in deaf patients with tinnitus and a cochlear implant [69]. In addition, it was shown that aversive sounds mimicking tinnitus presented to subjects without tinnitus also showed regional cerebral blood flow changes in the cerebellum [70].

A recent paper by Schecklmann and collaborators [71] could only find an uncorrected significant correlation between tinnitus related distress and a decrease in grey matter volume within the auditory cortex and insula after correction from potential cofounders such as age, gender, and audiometric parameters [71]. In contrast we found after correction for multiple comparisons only cerebellar changes survive statistical analysis, in contrast to the QEEG data in this study. The discrepancies between previous and current structural results could be related to the variability of clinical characteristics of the population studied. The results of this study indeed reveal that tinnitus lateralization, tinnitus distress, tinnitus loudness and tinnitus duration all influence the outcome. Therefore it is important to take these different characteristics into account when looking at structural (and functional) differences in the brain of tinnitus patients. Thus one should control for most characteristics important in tinnitus when analyzing a specific tinnitus characteristic, similar to what has been done for QEEG studies [15; 30; 32; 72]. However high interindividual anatomical variability for certain brain areas with respect to gyration and angulation may constitute a disadvantage for VBM approaches, = because interindividual anatomical variability is likely to translate into regionally fluctuating statistical power to detect group differences [73]. Thus, our data indicate that minor differences in populations with regard to tinnitus characteristics may account for differing results.

Another possibility for obtaining different results between labs might be different MRI systems (e.g. 1.5 vs. 3 Tesla) with different image acquisitions and data analysis. This explanation is unlikely as a previous study [20] tried to closely follow the protocol of another study [22] with only slight modifications and could not replicate the results even after lowering the statistical threshold, and defining specific regions of interest [20; 22]. Nevertheless, differences in machinery and acquisitions could contribute to differences in findings.

The question then is whether VBM is useful in evaluating functional pathologies such as tinnitus? Arguments against the use of VBM are the following: (1) Only the cerebellum survives statistically correct analysis by multiple comparisons, but not if controlled for hearing loss. (2) TMS of the auditory cortex in healthy controls evokes VBM changes without clinical changes [74], (3) It is not known what VBM really measures at a cellular level. (4) Between centres results are not reproducible, and (5) it remains to be elucidated by interventional studies whether the observed correlations reflect causal relations or are purely epiphenomena. Moreover, we analysed only the effect of selected clinical characteristics. Other clinically relevant factors may influence brain activity as well. The effects detected in the present study were rather moderate (liberal significance threshold, big sample size …).

Nevertheless, some arguments in favour of VBM can also be given. (1) Results in the different studies converge in the sense that the brain areas showing structural VBM changes occur in brain areas already implicated in tinnitus via functional imaging studies. (2) One centre has been able to replicate its own results [22; 23] (3) it is possible that tinnitus subgroups with similar characteristics might differ in their underlying neurobiological mechanism [75].

In most cases phantom sounds are the result of a lack of auditory input due to deafferentation as a consequence of a noise trauma or presbyacusis (i.e. age-related hearing loss) [10]. The VBM results obtained in previous research on tinnitus might not be directly related to tinnitus related factors, but by hearing loss as such. This supports the results of the Husain et al. (2011) where the most significant structural changes appeared in the group with hearing loss without tinnitus and there were no statistically significant differences between the tinnitus with hearing loss group and the normal hearing group. In addition, we showed that the effect of hearing loss was still present after performing regression analysis without specific tinnitus characteristics. Regression for the tinnitus characteristics without hearing loss generated no effect that survived correction. These findings were further confirmed by Melcher et al. (2012) revealing no statistical difference in grey matter comparing a tinnitus group with a control group, all with normal hearing at standard clinical frequencies (< 8 Hz). However, they did find a negative correlation between VBM and hearing thresholds at supra-clinical frequencies (< 8Hz). Previous research on structural changes in tinnitus evaluated a very heterogeneous group. While Mühlau et al. (2006) and Landgrebe et al. (2009) included no tinnitus patients with hearing loss of more than 25 dB HL nor did they report any kind of noise trauma, chronic, noise exposure or hyperacusis. Schneider et al. (2009) and Leaver et al. (2011) included tinnitus patients with a varying degree of hearing loss. This is further in line with several recent findings that have demonstrated structural changes due to aging or musical ability, but they did not mention tinnitus. That is, a relationship was revealed between the cortical neuroanatomy of cognitive brain regions and spoken word processing in the older adults [76]. Above that, increased gray matter volume of left pars opercularis in musicians correlated positively with years of musical performance [77].

Future research should confirm our findings using different functional imaging techniques such as positron emission tomography (PET), and magnetoencephalography (MEG), since QEEG has a poorer spatial resolution in comparison to those two. Nevertheless the advantages of using QEEG lie in its quiet operation, relatively inexpensive usage and better temporal resolution with respect to PET and fMRI. The sLORETA source analysis methodology has received considerable validation from studies combining LORETA with other more established localization methods, such as functional Magnetic Resonance Imaging (fMRI) [78; 79], and Positron Emission Tomography (PET) [80; 81; 82]. Further sLORETA validation has been based on findings obtained from invasive, implanted depth electrodes, in which case there are several studies in epilepsy [83; 84] and cognitive ERPs [85]. It is worth emphasizing that deep cortical structures such as the anterior cingulate cortex [86], and mesial temporal lobes [87] can be correctly localized with these methods. However, deeper structures such as the thalamus and cerebellum could not be included in our analysis due to the limitations of the source localized QEEG. As such the results should be interpreted with care. In addition, further research might also include secondary measure that might have an additional effect on the outcome such as hyperacusis, etc. as reported be in previous research [88].

In conclusion, functional changes as demonstrated by source localized QEEG are not reflected by associated structural changes. Specific tinnitus characteristics are also not reflected by structural changes. The VBM results obtained on tinnitus might not be directly related to tinnitus related factors, but instead to hearing loss.

## Supporting Information

S1 Fig The mean audiogram overall tinnitus patients. (DOCX)

S2 Fig Significant results for current density amplitude analysis in the alpha and beta frequency band. sLORETA current source density in the alpha1 (8–12 Hz) band correlated positively with tinnitus distress in the subgenual anterior cingulate, dorsal anterior cingulate and the hippocampus and in the beta (13–30 Hz) in the dorsal anterior cingulate cortex. This image shows significant results only.
(DOCX)

S3 Fig Significant results for current density amplitude analysis in the gamma frequency band. sLORETA current source density in the gamma (30.5–44 Hz) band correlated positively with tinnitus loudness in the left parahippocampal area. This image shows significant results only.
(DOCX)

S4 Fig Significant results for current density amplitude analysis in the theta frequency band. sLORETA current source density in the theta (4–7.5 Hz) band correlated positively with tinnitus duration in the dorsal anterior cingulate cortex area This image shows significant results only.
(DOCX)

S1 Table Local maxima from the different contrasts highlighting grey matter differences for tinnitus type, tinnitus lateralization, TQ, Vas loudness, Tinnitus duration, Tinnitus frequency and Tinnitus sensation level (N = 154). (DOCX)

S2 Table Split-half reliability analysis: Local maxima from the different contrasts highlighting grey matter differences for tinnitus type, tinnitus lateralization, TQ, Vas loudness, Tinnitus duration, Tinnitus frequency and Tinnitus sensation level (N = 154). (DOCX)

S3 Table Local maxima from the different contrasts highlighting grey matter differences for tinnitus type, tinnitus lateralization, TQ, Vas loudness, Tinnitus duration, Tinnitus frequency and Tinnitus sensation level (N = 154). (DOCX)

S1 Text Results integrative model for tinnitus (uncorrected). (DOCX)

S2 Text Results integrative model for tinnitus and hearing loss (uncorrected). (DOCX)
