# Supplementary material for: Tinnitus: A Large VBM-EEG Correlational Study
Source: PLoS One. 2015 Mar 17;10(3):e0115122. doi: 10.1371/journal.pone.0115122 (PMC4364116; doi:10.1371/journal.pone.0115122)
Supplement: S1 Fig — (DOCX) [file pone.0115122.s001.docx]

**Figure 1S. The mean audiogram overall tinnitus patients**
