# Supplementary material for: Tinnitus: A Large VBM-EEG Correlational Study
Source: PLoS One. 2015 Mar 17;10(3):e0115122. doi: 10.1371/journal.pone.0115122 (PMC4364116; doi:10.1371/journal.pone.0115122)
Supplement: S2 Fig — sLORETA current source density in the alpha1 (8–12 Hz) band correlated positively with tinnitus distress in the subgenual anterior cingulate, dorsal anterior cingulate and the hippocampus and in the beta (13–30 Hz) in the dorsal anterior cingulate cortex. This image shows significant results only. (DOCX) [file pone.0115122.s002.docx]

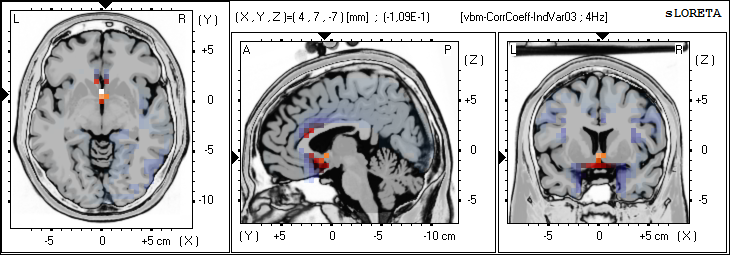


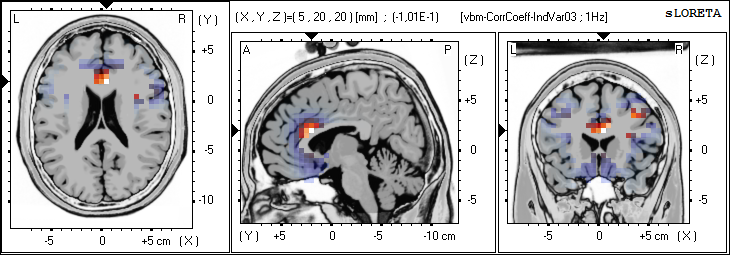


**Figure 2S. Significant results for current density amplitude analysis in the alpha and beta frequency band. sLORETA current source density in the alpha1 (8-12 Hz) band correlated positively with tinnitus distress in the subgenual anterior cingulate, dorsal anterior cingulate and the hippocampus and in the beta (13-30 Hz) in the dorsal anterior cingulate cortex . This image shows significant results only.**
