# Supplementary material for: Tinnitus: A Large VBM-EEG Correlational Study
Source: PLoS One. 2015 Mar 17;10(3):e0115122. doi: 10.1371/journal.pone.0115122 (PMC4364116; doi:10.1371/journal.pone.0115122)
Supplement: S3 Fig — sLORETA current source density in the gamma (30.5–44 Hz) band correlated positively with tinnitus loudness in the left parahippocampal area. This image shows significant results only. (DOCX) [file pone.0115122.s003.docx]

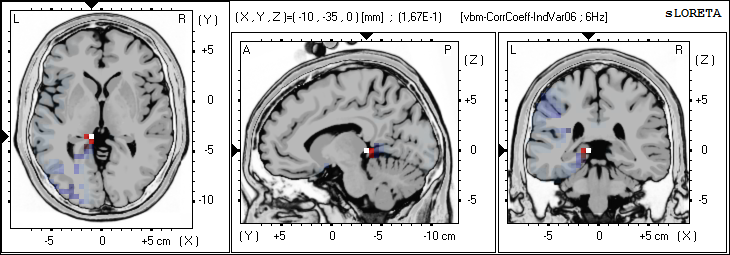


**Figure 3S. Significant results for current density amplitude analysis in the gamma frequency band. sLORETA current source density in the gamma (30.5-44 Hz) band correlated positively with tinnitus loudness in the left parahippocampal area. This image shows significant results only.**
