# Supplementary material for: Tinnitus: A Large VBM-EEG Correlational Study
Source: PLoS One. 2015 Mar 17;10(3):e0115122. doi: 10.1371/journal.pone.0115122 (PMC4364116; doi:10.1371/journal.pone.0115122)
Supplement: S4 Fig — sLORETA current source density in the theta (4–7.5 Hz) band correlated positively with tinnitus duration in the dorsal anterior cingulate cortex area This image shows significant results only. (DOCX) [file pone.0115122.s004.docx]

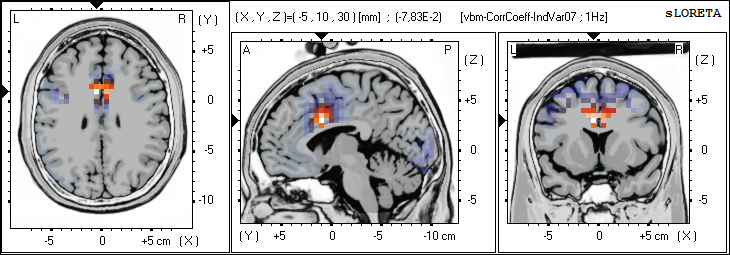


**Figure 4S. Significant results for current density amplitude analysis in the theta frequency band. sLORETA current source density in the theta (4-7.5 Hz) band correlated positively with tinnitus duration in the dorsal anterior cingulate cortex area This image shows significant results only.**
