# Supplementary material for: Tinnitus: A Large VBM-EEG Correlational Study
Source: PLoS One. 2015 Mar 17;10(3):e0115122. doi: 10.1371/journal.pone.0115122 (PMC4364116; doi:10.1371/journal.pone.0115122)
Supplement: S1 Table — (DOCX) [file pone.0115122.s005.docx]

**Table 1S. Local maxima from the different contrasts highlighting grey matter differences for tinnitus type, tinnitus lateralization, TQ, Vas loudness, Tinnitus duration, Tinnitus frequency and Tinnitus sensation level (N = 154).**

|  | | | **Coordinates** | | |  | **Significance** | |  | **Score** |  | **Cluster Size** |
| --- | --- | --- | --- | --- | --- | --- | --- | --- | --- | --- | --- | --- |
|  | | | MNI  x y z | | |  | *p* FDR corrected  at voxel level | *p* uncorrected |  | Z |  | Voxels |
| *1. Age* | | | | | | | | |  |  |  |  |
| + |  | |  |  |  |  |  |  |  |  |  |  |
|  | n.r.o. | |  |  |  |  |  |  |  |  |  |  |
| - |  | |  |  |  |  |  |  |  |  |  |  |
|  | n.r.o. | |  |  |  |  |  |  |  |  |  |  |
| *2. Gender* | | |  |  |  |  |  |  |  |  |  |  |
| + |  | |  |  |  |  |  |  |  |  |  |  |
|  | n.r.o. | |  |  |  |  |  |  |  |  |  |  |
| - |  | |  |  |  |  |  |  |  |  |  |  |
|  | n.r.o. | |  |  |  |  |  |  |  |  |  |  |
| *3. Type (NBN vs. PT)* | | | | | | | | |  |  |  |  |
| + |  | |  |  |  |  |  |  |  |  |  |  |
|  | n.r.o. | |  |  |  |  |  |  |  |  |  |  |
| - |  | |  |  |  |  |  |  |  |  |  |  |
|  | n.r.o. | |  |  |  |  |  |  |  |  |  |  |
| *4. Lateralization (Unilateral vs. Bilateral)* | | | | | | | | |  |  |  |  |
| + |  | |  |  |  |  |  |  |  |  |  |  |
|  | n.r.o. | |  |  |  |  |  |  |  |  |  |  |
| - |  | |  |  |  |  |  |  |  |  |  |  |
|  | Thalamus | R | -2 | -13 | -14 |  | .63 | < .001 |  | 3.27 |  | 23 |
|  | Hippocampus | L | -17 | -27 | -8 |  | .70 | .001 |  | 3.14 |  | 50 |
|  | Auditory cortex | R | 62 | -12 | -5 |  | .77 | .001 |  | 3.02 |  | 77 |
| *5. TQ (tinnitus related distress)* | | | | | | | | |  |  |  |  |
| + |  | |  |  |  |  |  |  |  |  |  |  |
|  | Parahippocampal | L | -11 | -10 | -35 |  | .37 | < .001 |  | 3.69 |  | 172 |
| - |  | |  |  |  |  |  |  |  |  |  |  |
|  | Cerebellum VIIb | L | -32 | -36 | -39 |  | .02 | < .001 |  | 4.29 |  | 464 |
|  |  | | -38 | -55 | -45 |  |  |  |  |  |  |  |
|  |  | | -50 | -73 | -30 |  |  |  |  |  |  |  |
|  | Crus II | R | 35 | -36 | -39 |  | .11 | < .001 |  | 3.94 |  | 204 |
|  |  | | 30 | -49 | -48 |  |  |  |  |  |  |  |
|  | Hippocampus | R | 18 | -30 | -9 |  | .12 | < .001 |  | 3.93 |  | 193 |
| *6.Tinnitus loudness* | | | | | | | | |  |  |  |  |
| + |  | |  |  |  |  |  |  |  |  |  |  |
|  | n.r.o. | |  |  |  |  |  |  |  |  |  |  |
| - |  | |  |  |  |  |  |  |  |  |  |  |
|  | Crus I | L | -62 | -52 | -26 |  | .03 | < .001 |  | 4.22 |  | 68 |
|  | Mid temporal | R | 53 | -1 | -20 |  | .40 | < .001 |  | 3.40 |  | 87 |
|  | Mid temporal | R | 59 | -22 | -6 |  | .46 | < .001 |  | 3.33 |  | 302 |
| *7. Duration* | | | | | | | | |  |  |  |  |
| + | | |  |  |  |  |  |  |  |  |  |  |
|  | n.r.o. | |  |  |  |  |  |  |  |  |  |  |
| - | | |  |  |  |  |  |  |  |  |  |  |
|  | Cerebellum X | R | 21 | -37 | -41 |  | .04 | < .001 |  | 4.25 |  | 703 |
|  |  |  | 29 | -36 | -45 |  |  |  |  |  |  |  |
|  | Parahippocampus | R | -17 | -18 | -11 |  | .37 | < .001 |  | 3.67 |  | 70 |
|  | Temporal Inferior | L | -59 | -63 | -23 |  | .44 | < .001 |  | 3.43 |  | 475 |
|  | Temporal Inferior | R | 60 | -58 | -23 |  | .50 | .001 |  | 3.33 |  | 71 |
| *8. Tinnitus Frequency* | | | | | | | | |  |  |  |  |
| + |  | |  |  |  |  |  |  |  |  |  |  |
|  | n.r.o. | |  |  |  |  |  |  |  |  |  |  |
| - |  | |  |  |  |  |  |  |  |  |  |  |
|  | n.r.o. | |  |  |  |  |  |  |  |  |  |  |
| *9. Tinnitus Sensation Level* | | | | | | | | |  |  |  |  |
| + |  | |  |  |  |  |  |  |  |  |  |  |
|  | n.r.o. | |  |  |  |  |  |  |  |  |  |  |
| - |  | |  |  |  |  |  |  |  |  |  |  |
|  | n.r.o. | |  |  |  |  |  |  |  |  |  |  |

n.r.o. = no results obtained; R: right; L: left
