# Supplementary material for: Tinnitus: A Large VBM-EEG Correlational Study
Source: PLoS One. 2015 Mar 17;10(3):e0115122. doi: 10.1371/journal.pone.0115122 (PMC4364116; doi:10.1371/journal.pone.0115122)
Supplement: S2 Table — (DOCX) [file pone.0115122.s006.docx]

**Table 2S. Split-half reliability analysis**: **Local maxima from the different contrasts highlighting grey matter differences for tinnitus type, tinnitus lateralization, TQ, Vas loudness, Tinnitus duration, Tinnitus frequency and Tinnitus sensation level (N = 154).**

|  | | | **Coordinates** | | |  | **Significance** | |  | **Score** |  | **Cluster Size** |
| --- | --- | --- | --- | --- | --- | --- | --- | --- | --- | --- | --- | --- |
|  | | | MNI  x y z | | |  | *p* FDR corrected  at voxel level | *p* uncorrected |  | Z |  | Voxels |
| *Group 1 (=77)* | | | | | | | | |  |  |  |  |
| *1. TQ (tinnitus related distress)* | | | | | | | | |  |  |  |  |
| - |  | |  |  |  |  |  |  |  |  |  |  |
|  | Cerebellum IX | L | 0 | -42 | -41 |  | .26 | .001 |  | 3.74 |  | 6 |
|  |  | L | -5 | -33 | -23 |  |  |  |  |  |  |  |
|  |  | R | 8 | -31 | -21 |  |  |  |  |  |  |  |
| *2.Tinnitus loudness* | | | | | | | | |  |  |  |  |
| - |  | |  |  |  |  |  |  |  |  |  |  |
|  | Cerebellum IX | L | 29 | 9 | -47 |  | .42 | .000 |  | 2.24 |  | 452 |
|  | Mid temporal Pole | R | -11 | -43 | -42 |  | .65 | .019 |  | 2.08 |  | 340 |
|  | Hippocampus | R | 24 | -25 | -11 |  | .94 | .026 |  | 1.94 |  | 58 |
| *3. Duration* | | | | | | | | |  |  |  |  |
| - | | |  |  |  |  |  |  |  |  |  |  |
|  | Crus I | R | 53 | -57 | -24 |  | .36 | .000 |  | 3.06 |  | 347 |
|  | Cerebellum VIII | R | 39 | -44 | -51 |  | .78 | .000 |  | 3.94 |  | 126 |
|  | Inferior Temporal | R | -18 | -24 | -14 |  | .58 | .007 |  | 2.46 |  | 167 |
| *Group 2 (=77)* | | | | | | | | |  |  |  |  |
| *1. TQ (tinnitus related distress)* | | | | | | | | |  |  |  |  |
| - |  | |  |  |  |  |  |  |  |  |  |  |
|  | Crus I | L | -50 | -55 | -27 |  | .96 | .05 |  | 1.79 |  | 6 |
| *2.Tinnitus loudness* | | | | | | | | |  |  |  |  |
| - |  | |  |  |  |  |  |  |  |  |  |  |
|  | Cerebellum IX | R | 12 | -58 | -39 |  | .40 | .000 |  | 2.49 |  | 42 |
|  |  |  | 12 | -46 | -33 |  |  |  |  |  |  |  |
| *3. Duration* | | | | | | | | |  |  |  |  |
| - | | |  |  |  |  |  |  |  |  |  |  |
|  | Hippocampus | R | 8 | -10 | -17 |  | .57 | .000 |  | 3.68 |  | 207 |

R: right; L: left
