# Supplementary material for: Tinnitus: A Large VBM-EEG Correlational Study
Source: PLoS One. 2015 Mar 17;10(3):e0115122. doi: 10.1371/journal.pone.0115122 (PMC4364116; doi:10.1371/journal.pone.0115122)
Supplement: S3 Table — (DOCX) [file pone.0115122.s007.docx]

**Table 3S. Local maxima from the different contrasts highlighting grey matter differences for tinnitus type, tinnitus lateralization, TQ, Vas loudness, Tinnitus duration, Tinnitus frequency and Tinnitus sensation level (N = 154).**

|  | | | **Coordinates** | | |  | **Significance** | |  | **Score** |  | **Cluster Size** |
| --- | --- | --- | --- | --- | --- | --- | --- | --- | --- | --- | --- | --- |
|  | | | MNI  x y z | | |  | *p* FDR corrected  at voxel level | *p* uncorrected |  | Z |  | Voxels |
| *1. Age* | | | | | | | | |  |  |  |  |
| + |  | |  |  |  |  |  |  |  |  |  |  |
|  | n.r.o. | |  |  |  |  |  |  |  |  |  |  |
| - |  | |  |  |  |  |  |  |  |  |  |  |
|  | n.r.o. | |  |  |  |  |  |  |  |  |  |  |
| *2. Gender* | | |  |  |  |  |  |  |  |  |  |  |
| + |  | |  |  |  |  |  |  |  |  |  |  |
|  | n.r.o. | |  |  |  |  |  |  |  |  |  |  |
| - |  | |  |  |  |  |  |  |  |  |  |  |
|  | n.r.o. | |  |  |  |  |  |  |  |  |  |  |
| *3. Type (NBN vs. PT)* | | | | | | | | |  |  |  |  |
| + |  | |  |  |  |  |  |  |  |  |  |  |
|  | n.r.o. | |  |  |  |  |  |  |  |  |  |  |
| - |  | |  |  |  |  |  |  |  |  |  |  |
|  | n.r.o. | |  |  |  |  |  |  |  |  |  |  |
| *4. Lateralization (Unilateral vs. Bilateral)* | | | | | | | | |  |  |  |  |
| + |  | |  |  |  |  |  |  |  |  |  |  |
|  | n.r.o. | |  |  |  |  |  |  |  |  |  |  |
| - |  | |  |  |  |  |  |  |  |  |  |  |
|  | n.r.o. |  |  |  |  |  |  |  |  |  |  |  |
| *5. TQ (tinnitus related distress)* | | | | | | | | |  |  |  |  |
| + |  | |  |  |  |  |  |  |  |  |  |  |
|  | n.r.o. |  |  |  |  |  |  |  |  |  |  |  |
| - |  | |  |  |  |  |  |  |  |  |  |  |
|  | n.r.o. |  |  |  |  |  |  |  |  |  |  |  |
| *6.Tinnitus loudness* | | | | | | | | |  |  |  |  |
| + |  | |  |  |  |  |  |  |  |  |  |  |
|  | n.r.o. | |  |  |  |  |  |  |  |  |  |  |
| - |  | |  |  |  |  |  |  |  |  |  |  |
|  | n.r.o. |  |  |  |  |  |  |  |  |  |  |  |
| *7. Duration* | | | | | | | | |  |  |  |  |
| + | | |  |  |  |  |  |  |  |  |  |  |
|  | n.r.o. | |  |  |  |  |  |  |  |  |  |  |
| - | | |  |  |  |  |  |  |  |  |  |  |
|  | n.r.o. |  |  |  |  |  |  |  |  |  |  |  |
| *8. Tinnitus Frequency* | | | | | | | | |  |  |  |  |
| + |  | |  |  |  |  |  |  |  |  |  |  |
|  | n.r.o. | |  |  |  |  |  |  |  |  |  |  |
| - |  | |  |  |  |  |  |  |  |  |  |  |
|  | n.r.o. | |  |  |  |  |  |  |  |  |  |  |
| *9. Tinnitus Sensation Level* | | | | | | | | |  |  |  |  |
| + |  | |  |  |  |  |  |  |  |  |  |  |
|  | n.r.o. | |  |  |  |  |  |  |  |  |  |  |
| - |  | |  |  |  |  |  |  |  |  |  |  |
|  | n.r.o. | |  |  |  |  |  |  |  |  |  |  |
| *10. Hearing loss* | | | | | | | |  |  |  |  |  |
| + |  | |  |  |  |  |  |  |  |  |  |  |
|  | n.r.o. | |  |  |  |  |  |  |  |  |  |  |
| - |  | |  |  |  |  |  |  |  |  |  |  |
|  | Auditory Cortex | L | -29 | -36 | 19 |  | .007 | < .001 |  | 4.86 |  | 1850 |
|  | Thalamus | L | 0 | -6 | 21 |  | .02 | < .001 |  | 4.77 |  | 808 |
|  | Ventral lateral prefrontal cortex |  | -18 | 32 | -8 |  | .03 |  |  | 4.57 |  | 492 |
|  | Caudate Nucleus |  | -15 | 29 | 10 |  | .04 | < .001 |  | 4.33 |  | 341 |
|  | Crus I | L | -56 | -63 | -32 |  | .04 | < .001 |  | 3.72 |  | 125 |
|  | Crus II | R | 59 | -53 | -39 |  | .04 | .001 |  | 3.51 |  | 75 |

n.r.o. = no results obtained; R: right; L: left
