# Supplementary material for: Tinnitus: A Large VBM-EEG Correlational Study
Source: PLoS One. 2015 Mar 17;10(3):e0115122. doi: 10.1371/journal.pone.0115122 (PMC4364116; doi:10.1371/journal.pone.0115122)
Supplement: S1 Text — (DOCX) [file pone.0115122.s008.docx]

1. Integrative model: tinnitus

Uncorrected, a regression analysis further demonstrated significant effects for tinnitus lateralization, tinnitus distress, tinnitus loudness and tinnitus duration (see Table 2S for overview). For tinnitus lateralization it was shown that the thalamus (Z = 3.27, *p_uncorrected_* < .001), the left hippocampus (Z = 3.14, *p_uncorrected_* = .001) and the right primary auditory cortex (Z = 3.02, *p_uncorrected_* = .001) had a smaller grey matter density for the unilateral tinnitus patients in comparison to the bilateral tinnitus patients (see Figure 1A). For tinnitus related distress it was shown that the left parahippocampal area (Z = 3.69, *p_uncorrected_* < .001) showed increased grey matter concentration in association with more distress as measured with the tinnitus questionnaire (see Figure 1B). However, the opposite was found for the left cerebellum VIIIb (Z = 4.29, *p_uncorrected_* < .001), right crus II (Z = 3.94, *p_uncorrected_* < .001) and right hippocampus (Z = 3.93, *p_uncorrected_* < .001), indicating the grey matter concentration decreased in association with more distress as measured with the tinnitus questionnaire (see Figure 1C). For tinnitus loudness decreased grey matter density goes together with increased subjective tinnitus loudness within the crus I (Z = 4.22, *p_uncorrected_* < .001) and the right mid temporal brain area (Z = 3.40, *p_uncorrected_* < .001) (see Figure 1D). In addition, it was shown that the cerebellum X (Z = 4.25, *p_uncorrected_* < .001), right parahippocampus (Z = 3.67, *p_uncorrected_* < .001), inferior termporal brain area (Z = 3.43, *p_uncorrected_* < .001) showed decreased grey matter density in association with longer tinnitus duration (see Figure 1E).
