# Supplementary material for: Tinnitus: A Large VBM-EEG Correlational Study
Source: PLoS One. 2015 Mar 17;10(3):e0115122. doi: 10.1371/journal.pone.0115122 (PMC4364116; doi:10.1371/journal.pone.0115122)
Supplement: S2 Text — (DOCX) [file pone.0115122.s009.docx]

2. Integrative model: Tinnitus and hearing loss

A regression analysis revealed several uncorrected significant effect was hearing loss (see Table 3S, Figure 1F for overview). It was shown that the auditory cortex (Z = 4.86, *p_uncorrected_* < .001), thalumus (Z = 4.77, *p_uncorrected_* = .001), ventrolateral prefrontal cortex (Z = 4.57, *p_uncorrected_* < .001), the caudate nucleus (Z = 4.33, *p_uncorrected_* < .001), Crus I (Z = 3.72, *p_uncorrected_* < .001) and Crus II (Z = 3.51, *p_uncorrected_* = .001) had a smaller grey matter density for the tinnitus patients with more hearing loss.
